# Clinical significance of PNO1 as a novel biomarker and therapeutic target of hepatocellular carcinoma

**DOI:** 10.1111/jcmm.18295

**Published:** 2024-05-09

**Authors:** Sanjit K. Roy, Shivam Srivastava, Caroline McCance, Anju Shrivastava, Jason Morvant, Sharmila Shankar, Rakesh K. Srivastava

**Affiliations:** ^1^ Stanley S. Scott Cancer Center, School of Medicine Louisiana State University Health New Orleans Louisiana USA; ^2^ Louisiana State University Baton Rouge Louisiana USA; ^3^ Department of Cellular and Molecular Biology Tulane University New Orleans Louisiana USA; ^4^ St. Joseph's Hospital and Medical Center Phoenix Arizona USA; ^5^ Department of Surgery Ochsner Health System Gretna Louisiana USA; ^6^ Southeast Louisiana Veterans Health Care System New Orleans Louisiana USA; ^7^ John W. Deming Department of Medicine Tulane University School of Medicine New Orleans Louisiana USA; ^8^ Department of Genetics Louisiana State University Health Sciences Center – New Orleans New Orleans Louisiana USA; ^9^ GLAX Dover Delaware USA

**Keywords:** biogenesis, cancer stem cell, CRISPR/Cas9, hepatocellular carcinoma, notch, PNO1

## Abstract

The RNA‐binding protein PNO1 plays an essential role in ribosome biogenesis. Recent studies have shown that it is involved in tumorigenesis; however, its role in hepatocellular carcinoma (HCC) is not well understood. The purpose of this study was to examine whether PNO1 can be used as a biomarker of HCC and also examine the therapeutic potential of PNO1 knockout for the treatment of HCC. PNO1 expression was upregulated in HCC and associated with poor prognosis. PNO1 expression was positively associated with tumour stage, lymph node metastasis and poor survival. PNO1 expression was significantly higher in HCC compared to that in fibrolamellar carcinoma or normal tissues. Furthermore, HCC tissues with mutant Tp53 expressed higher PNO1 than those with wild‐type Tp53. PNO1 knockout suppressed cell viability, colony formation and EMT of HCC cells. Since activation of Notch signalling pathway promotes HCC, we measured the effects of PNO1 knockout on the components of Notch pathway and its targets. PNO1 knockout suppressed Notch signalling by modulating the expression of Notch ligands and their receptors, and downstream targets. PNO1 knockout also inhibited genes involved in surface adhesion, cell cycle, inflammation and chemotaxis. PNO1 knockout also inhibited colony and spheroid formation, cell migration and invasion, and markers of stem cells, pluripotency and EMT in CSCs. Overall, our data suggest that PNO1 can be used as a diagnostic and prognostic biomarker of HCC, and knockout of PNO1 by CRISPR/Cas9 can be beneficial for the management of HCC by targeting CSCs.

## 1 INTRODUCTION

Hepatocellular carcinoma (HCC) is a leading cause of cancer‐related mortality and morbidity worldwide.^1^ HCC is the most common form of liver cancer and accounts for about 90% of cases. In the United States, the incidence of HCC has more than quadrupled, becoming the fastest‐growing cause of cancer‐related deaths. According to the American Cancer Society, the HCC is expected to become the third leading cause of cancer‐related mortality by 2030.^1^ Current standard therapies are not curative for advanced disease and minimally improve survival; hence, there is an unmet need to identify new prognostic molecular biomarkers and develop novel molecular targeted therapy to combat this devastating disease.

In eukaryotes, ribosomes act as molecular machinery and are responsible for the translation of mRNA into protein. The RNA‐binding protein PNO1 is a ribosome assembly factor that plays an essential role in ribosome biogenesis.^2^, ^3^ It is located in human chromosome 2p14, comprising seven exons and six introns. It is highly conserved from yeast to mammals. PNO1 cleaves 18S mediated by binding to NOB1.^4^ The dysregulated ribosome biogenesis has been demonstrated during the initiation and progression of spontaneous cancers.^5^, ^6^ An increased rate of ribosomal biogenesis is positively correlated with increased size and numbers of nucleoli in cancer development. In addition to ribosome biogenesis, it also regulates cancer progression by modulating autophagy‐mediated apoptosis or ferroptosis.^7^, ^8^, ^9^, ^10^ Despite these functions, the mechanism by which PNO1 regulates HCC is not well understood. Since PNO1 is highly expressed in several cancers, it can be used as a biomarker and targeted for the effective treatment of HCC.

The Notch pathway transmits its signal by direct cell‐to‐cell interaction in multicellular organisms.^11^, ^12^, ^13^ Dysregulated Notch signalling pathway is responsible for abnormal cell growth, development, differentiation and apoptosis.^11^, ^14^, ^15^, ^16^ The Notch signalling pathway consists of five ligands which are Jagged‐1, Jagged‐2, Delta‐like‐1 (Dll1), Delta‐like‐3 (Dll‐3) and Delta‐Like‐4 (Dll‐4).^14^, ^17^ By comparison, this pathway transmits its signal through four receptors namely Notch‐1, Notch‐2, Notch‐3 and Notch‐4.^14^, ^17^ While the Notch ligands are the single‐pass transmembrane proteins of DSL family, the Notch receptors are transmembrane proteins containing both types of extracellular and intracellular domains.^14^ The binding of ligand to the various Notch receptors leads to a conformational change resulting in ADAM‐mediated ectodomain shedding and subsequent γ‐secretase‐mediated proteolysis of the receptor. Sequential occurrence of these events results in the release of Notch intracellular domain (NICD).^18^ Following proteolysis, NICD translocates to the nucleus and associates with the DNA‐binding protein CSL/RBPjκ, where the coactivators (MAML, p300 and HAT) bind together to activate the transcriptional complex and induce genes such as families of Hairy/Enhancer of Split (HES) and Hairy/Enhancer of Split related to YRPW motif (HEY). Recent studies have demonstrated the oncogenic role of Notch in HCC,^19^, ^20^, ^21^ suggesting the inhibition of the Notch signalling pathway can be exploited for the management of HCC.

In this paper, we examine whether PNO1 can be used as a biomarker for HCC and examine the molecular mechanisms by which PNO1 inhibition regulates HCC growth and epithelial‐mesenchymal transition (EMT). We have used CRISPR/Cas9 technology to knockout PNO1 expression. Our data demonstrated that the expression of PNO1 was significantly higher in HCC than those in normal tissue, suggesting it can be used as a diagnostic biomarker of HCC. PNO1 knockout inhibited growth and EMT of HCC cells. Higher PNO1 expression was associated with the poor survival of HCC patients. Furthermore, PNO1 knockdown inhibited the oncogenic Notch signalling pathway, which plays a crucial role in HCC development. PNO1 knockout also inhibited CSC characteristics by suppressing stem cell markers and pluripotency‐maintaining factors. In conclusion, PNO1 can be used as a diagnostic and prognostic biomarker of HCC, and knockout of PNO1 by CRISPR/Cas9 can be used for the treatment of HCC by targeting CSCs.

## 2 MATERIALS AND METHODS

### 2.1 Reagents

Antibodies against CD44 (cat # 5640), CD133 (Cat #51917) and AFP (Cat #43035) were purchased from Cell Signaling Technology (Danvers, MA). Antibody against PNO1 (SC‐514727) was purchased from Santa Cruz Biotechnology, Inc. (Dallas, Texas). Dulbecco's Modified Eagle's Medium (DMEM), foetal bovine serum (FBS), penicillin and streptomycin were purchased from Thermo Fisher Scientific (Suwanee, GA). All other chemicals were purchased from Sigma‐Aldrich (St. Louis, MO). Lentiviral vectors containing either non‐targeting control (CCPCTR01‐LvSG03‐B 3xsg RNA lentiviral expression clone) or PNO1 CRISPR/Cas9 [HCP307322‐LvSG03‐3 3xsgRNA lentiviral exp clone targeting PNO1 (NM_001329916.1); CP‐LvC9NU‐02 Cas9 nuclease lentiviral expression clone] were purchased from GeneCopoeia (Rockville, MD).

### 2.2 Cell culture

Hep3B, HepG2 and HEK293T cells were purchased from American Type Culture Collection (ATCC), Manassas, VA, and were authenticated by the vendor using short tandem repeat (STR) profiling. Qiu et al has described the genetic and pharmacological differences between Hep3B and HepG2 cell lines.^22^ HepG2 cell line was isolated from liver biopsy specimens of a 15‐year‐old Caucasian male from Argentina with primary hepatoblastoma.^23^ Hep3B cell line was isolated from an 8‐year‐old black male from the United States of America with primary HCC.^24^ HepG2 cells contain an average of 55 (50–56) chromosomes per cell whereas Hep3B cells, 60.^22^ In addition, HepG2 is hepatitis B virus negative and non‐tumorigenic, but Hep3B is hepatitis B virus positive and tumorigenic.^24^, ^25^

HCC cell lines were grown in Dulbecco's Modified Eagle's Medium (DMEM) which was supplemented with 10% v/v FBS, 1% v/v L‐glutamine, 100 U/mL penicillin and 100 μg/mL streptomycin. All the cell lines were maintained at 37°C in 5% CO_2_ at constant humidity. CD44^+^/CD133^+^ human cancer stem cells (CSCs) were isolated from primary hepatocellular carcinoma (obtained from Celprogen, Torrance, CA) and grown in a well‐defined stem cell culture medium, as per the supplier's instructions.

### 2.3 Lentiviral particle production and transduction

The protocol for lentivirus production and transduction have been described elsewhere.^26^ Briefly, HEK293T cells were transfected with 4 μg of plasmid and 4 μg of the lentiviral vectors using Lipofectamine‐3000 according to the manufacturer's protocol (Invitrogen). PEG‐it virus precipitation solution (SBI System Biosciences) was added to supernatant, and ultracentrifugation was performed to collect concentrated viral particles. HCC cells and CSCs were transduced with lentiviral particles with 6 μg/mL polybrene (Invitrogen).

### 2.4 Cell viability assay

Cell viability was measured as we described elsewhere.^27^ In brief, cells (1.5 × 10^4^) transduced with either non‐targeting control (NTC) or PNO1 CRISPR/Cas9 viral particles were grown in a cell culture medium for various time points. Cell viability was measured by CellTiter‐Glo® Luminescent Cell Viability (Promega) assay according to the manufacturer's instruction.

### 2.5 Spheroid assay

Spheroid formation assays were performed as described elsewhere.^26^ Briefly, CSCs were plated in 6‐well ultralow attachment plates (Corning Inc., Corning, NY) at a density of 100 to 500 cells/mL in stem cell growth medium with 1% N2 Supplement (Invitrogen), 2% B27 Supplement (Invitrogen), 20 ng/mL human platelet growth factor (Sigma‐Aldrich), 100 ng/mL epidermal growth factor (Invitrogen) and 1% antibiotic‐antimycotic (Invitrogen) at 37°C in a humidified atmosphere of 95% air and 5% CO_2_. Spheroids were collected after 7 days and dissociated with Accutase (Innovative Cell Technologies, Inc.). The CSCs obtained from dissociation were counted using trypan blue dye.

### 2.6 Motility assay

Cell motility assay was performed as we described elsewhere.^28^, ^29^ In brief, cells (non‐targeting control, NTC and CRISPR/Cas9) were grown to a confluent monolayer in a 6‐well plate, scratched with a 200‐μL tip and washed twice with PBS. Liver cancer cells were grown in cell culture medium and photographed at 0, and 48 h under an inverted microscope. The width of the scratch gap is viewed under the microscope in several separate areas until the gap is filled in the untreated control wells.

### 2.7 Transwell migration assay

Transwell migration assays were performed according to the protocol we described elsewhere.^30^ In brief, 1 × 10^5^ cells (non‐targeting control, NTC and CRISPR/Cas9) in 200 μL of medium with 1% FBS were plated in the top chamber onto the non‐coated membrane (6.5‐mm diameter, 8‐μm pores; Corning Costar, Corning, NY) and allowed to migrate in the lower chamber towards 10% FBS (as chemoattractant)‐containing medium. Cells were incubated for 48 h at 37°C in 5% CO_2_. Subsequently. cells were fixed with methanol, stained with crystal violet and counted under an inverted microscope.

### 2.8 Transwell invasion assay

Transwell invasion assays were performed according to the protocol we described elsewhere.^31^, ^32^ In brief, 1 × 10^5^ cells (non‐targeting control, NTC and CRISPR/Cas9) in 200 μL of medium with 1% FBS were plated on top of a layer of Matrigel in transwell chambers (6.5‐mm diameter, 8‐μm pores; Corning Costar, Corning, NY). Cells were allowed to invade the lower chamber towards 10% FBS (as chemoattractant)‐containing medium. After 72 h of incubation at 37°C in 5% CO_2_, cells that did not migrate were removed from the top of the transwell filters by scraping. Cells that had invaded the Matrigel were fixed with methanol, stained with crystal violet and counted under an inverted microscope.

### 2.9 Total RNA extraction

For total RNA extraction, the cells were pelleted and lysed with the TRIzol reagent. After incubation of samples for 5 min at room temperature, 200 μL of chloroform was added and centrifuged at 9000 × *g* for 15 min at 4°C. The aqueous phase was transferred to a new tube, and 500 μL of isopropanol was added, mixed and incubated for 10 min at room temperature. After centrifugation at 9000 × *g* for 10 min at 4°C, the supernatant was removed, and the pellet was washed with 1 mL of 70% ethanol and centrifuged at 5000 × *g* for 10 min at 4°C. Finally, the RNA pellet was air dried for 5 to 10 min, dissolved in 20 μL DEPC‐treated water and quantified with a NanoDrop™ 2000 spectrophotometer (Thermo Fisher Scientific).

### 2.10 Quantitative real‐time PCR

qRT‐PCR was performed as described elsewhere.^29^, ^33^ Briefly, cDNA was synthesized using a high‐capacity cDNA reverse transcription kit (Applied Biosystems). Primers specific for each of the signalling molecules were designed using NCBI/Primer‐BLAST and used to generate the PCR products. For the quantification of gene amplification, real‐time PCR was performed using an ABI 7300 Sequence Detection System in the presence of SYBR‐Green. The relative mRNA expression levels were determined using the ∆∆*C*_t_ method. The expression levels of target genes were normalized to the reference gene.

### 2.11 Statistical analysis

All the data represent the mean ± SD of at least three replicates. Differences between groups were analysed by ANOVA or *t*‐tests using PRISM statistical analysis software (GrafPad Software, Inc., San Diego, CA). The significant level of each experiment was reported at *p* < 0.05.

## 3 RESULTS

### 3.1 Differential expression of PNO1, which changes with stage, nodal metastasis, grade, histological subtype and Tp53 mutant status in hepatocellular carcinoma (HCC)

**FIGURE 1:**
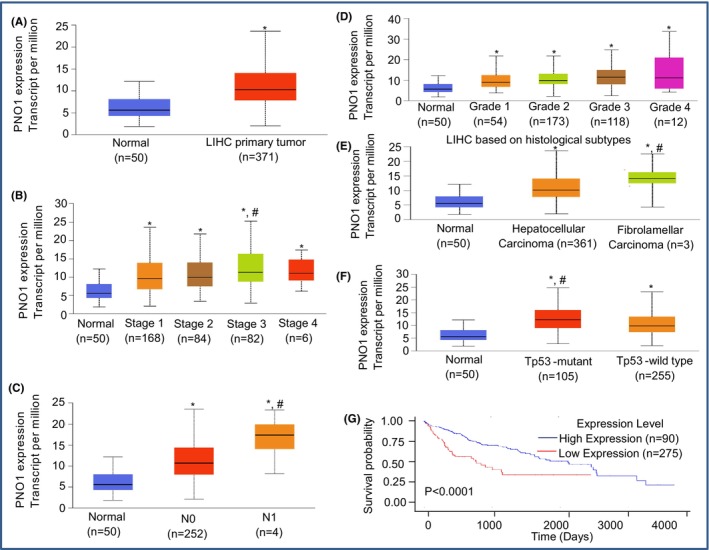
Differential expression of PNO1 which changes with stage, nodal metastasis, grade and Tp53 status in HCC. (A) PNO1 expression in HCC and normal liver tissues. PNO1 expression data were analysed using the Cancer Genome Atlas (TCGA) data bank by UALCAN (The University of Alabama at Birmingham Cancer Data Analysis Portal). Normal (*n* = 50), LIHC primary tumour (*n* = 371), * = significantly different from normal (*p* < 0.05). (B) PNO1 expression during various stages of HCC. Normal tissues (*n* = 50), Stage 1 (*n* = 168), Stage 2 (*n* = 84), Stage 3 (*n* = 82), Stage 4 (*n* = 6), *, # = significantly different from normal and each other (*p* < 0.05). (C), PNO1 expression at different stages of nodal metastasis. Normal (*n* = 50), N0 (*n* = 252), N1 (*n* = 4), *, # = significantly different from normal and each other (*p* < 0.05). (D) PNO1 expression during different tumour grades of HCC. Normal (*n* = 50), Grade 1 (*n* = 54), Grade 2 (*n* = 173), Grade 3 (*n* = 118), Grade 4 (*n* = 12), * = significantly different from normal and each other (*p* < 0.05). (E) PNO1 expression in different histological subtypes. Normal (*n* = 50), hepatocellular carcinoma (*n* = 361), fibrolamellar carcinoma (*n* = 3), *, # = significantly different from normal and each other (*p* < 0.05). (F) PNO1 expression in HCC with mutant and wild‐type Tp53 status. Normal (*n* = 50), Tp53‐mutant (*n* = 105) and Tp53‐wild type (*n* = 255), *, # = significantly different from normal and each other (*p* < 0.05). (G) Effects of PNO1 expression on survival probability of HCC patients. PNO1 high expression (*n* = 90) and PNO1 low expression (*n* = 275). Survival probability is significantly different between PNO1 high expression and low expression groups at *p* < 0.0001.

We first examined the expression of PNO1 in HCC using the Cancer Genome Atlas (TCGA) data bank by UALCAN (The University of Alabama at Birmingham Cancer Data Analysis Portal). The expression of PNO1 was significantly higher in HCC than in normal liver tissues (Figure 1A). We next assessed the associations of PNO1 expression with various clinicopathological factors in HCC (Figure 1B). PNO1 expression was significantly higher in all stages (stages 1–4) of HCC compared to normal tissues. The highest expression of PNO1 was observed in Stage 3. We next examined the PNO1 expression at different stages of nodal metastasis. PNO1 expression was significantly higher at N0 and N1 stages of nodal metastasis compared to normal tissues (Figure 1C). The highest expression of PNO1 was observed at N1 nodal metastasis. We then examined whether the expression of PNO1 changed during various grades of HCC (Figure 1D). PNO1 expression was significantly higher in all grades (grades 1–4) of HCC compared to normal tissues. The highest expression of PNO1 was observed in Grade 3. We next examined the PNO1 expression at different histological subtype (hepatocellular carcinoma and fibrolamellar carcinoma). PNO1 expression was significantly higher in both hepatocellular carcinoma and fibrolamellar carcinoma compared to that in normal tissues (Figure 1E). The maximum expression of PNO1 was observed in fibrolamellar carcinoma. TCGA data indicated that PNO1 expression was upregulated in HCC samples and was associated with advanced TNM stages and lymph node metastasis.

Tp53 is a transcription factor and also acts as a tumour suppressor gene. We next examined whether Tp53 mutant status plays a significant role on the expression of PNO1 in HCC (Figure 1F). PNO1 expression was significantly higher in both Tp53 mutant and wild‐type HCC compared to normal liver tissues. However, HCC tissues expressing mutant Tp53 showed significantly higher PNO1 expression than those expressing wild‐type Tp53. These data suggest that HCC patients harbouring mutant Tp53 may develop more aggressive tumours than those harbouring wild‐type Tp53.

We subsequently examined the PNO1 expression and survival rate of HCC patients. The survival curve showed that higher PNO1 expression was related with the poor survival of HCC patients. These data suggest that PNO1 expression might serve as a specific prognostic biomarker in HCC patients.

### 3.2 PNO1 knockout inhibits colony formation and cell proliferation in HCC

**FIGURE 2:**
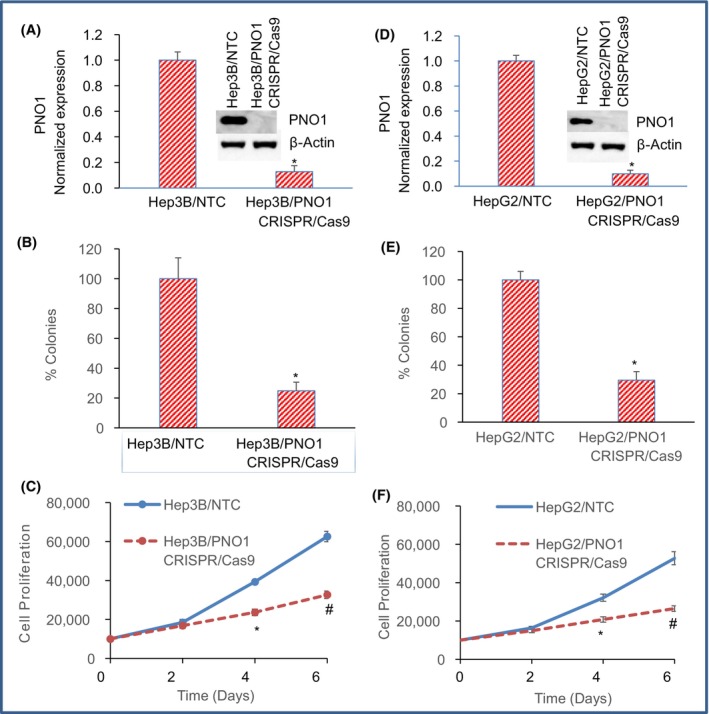
PNO1 knockout inhibits colony formation and cell proliferation in HCC. (A, D) PNO1 expression. Liver cancer (Hep3B and HepG2) cells were transduced with lentiviral particles expressing either non‐targeting control (NTC) or PNO1 CRISPR/Cas9, and the expression of PNO1 gene and protein was measured by qRT‐PCR and western blot analysis, respectively. (B, E) % Colony formation. Liver cancer (Hep3B and HepG2) cells were transduced with lentiviral particles expressing either non‐targeting control (NTC) or PNO1 CRISPR/Cas9. Number of colonies formed at 21 days were counted. Data represent mean (*n* = 4) ± SD. * = significantly different from NTC, *p* < 0.05. (C, F) Cell proliferation. Hep3B and HepG2 cells were transduced with lentiviral particles expressing either non‐targeting control (NTC) or PNO1 CRISPR/Cas9. Cell proliferation was measured at 0, 2, 4 and 6 days. Data represent mean (*n* = 4) ± SD. * = significantly different from NTC, *p* < 0.05.

PNO1 has been shown to positively regulate cancer progression^7^, ^8^, ^34^; we, therefore, examined the effects of PNO1 inhibition on growth of HCC cells (Hep3B and HepG2). The PNO1 expression was inhibited by CRISPR/Cas9 technology. Hep3B and HepG2 cells were transduced with lentiviral particles expressing either non‐targeting (NTC) control or PNO1 CRISPR/Cas9, and the expressions of PNO1 gene and protein were measured by qRT‐PCR and western blot analyses, respectively. PNO1 knockdown inhibited the expressions of both PNO1 gene and protein in Hep3B and HepG2 cells compared to NTC (Figure 2A,D).

Colony formation and cell proliferation assays are generally used to assess the effectiveness of cancer therapeutics. Since PNO1 knockout inhibited the expression of PNO1, we sought to examine the effects of inhibiting PNO1 on colony formation by HCC cells. The number of colonies formed was counted on Day 21. PNO1 knockout significantly inhibited colony formation in both Hep3B and HepG2 cells (Figure 2B,E). We next sought to examine the effects of inhibiting PNO1 on Hep3B and HepG2 cell proliferation (Figure 2C,F). PNO1 knockout significantly inhibited cell proliferation of both Hep3B and HepG2 cells at 2, 4 and 6 days. These data suggest that excessive PNO1 expression acts as oncogene and its knockout by CRISPR/Cas9 technique can be used for the treatment of HCC.

### 3.3 PNO1 knockout inhibits cell motility, migration and invasion of HCC

**FIGURE 3:**
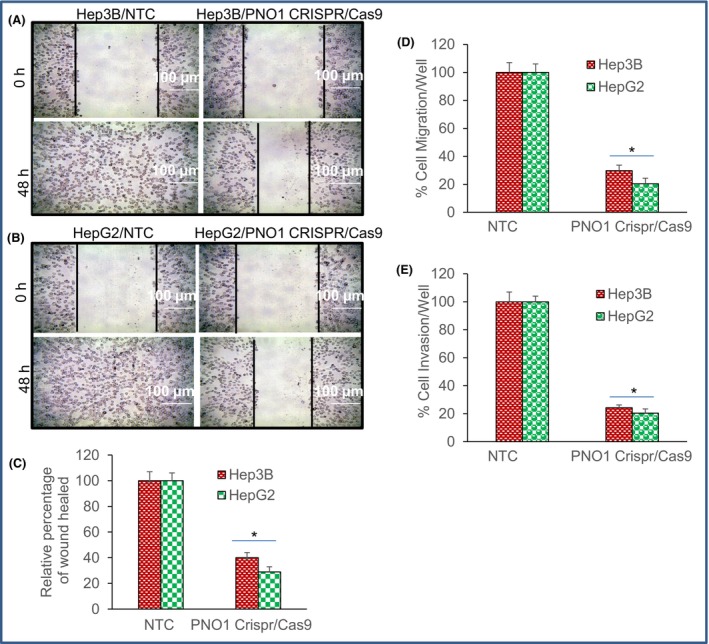
PNO1 knockout inhibits cell motility, migration and invasion of liver cancer cells. (A, B) Cell motility assay. Liver cancer cells (Hep3B and HepG2) expressing either non‐targeting control (NTC) or PNO1 CRISPR/Cas9 were grown in monolayer and scratched. Cells were photographed at 48 h. Images were obtained with 100× magnification. Images are captured at different time intervals as indicated. Graph shows a quantitative representation of distance remaining in the original scratch (width) as cells migrate over time. (C) Relative percentage of wound healed. Scratched distance at time 0 and 48 h was measured in NTC and CRISPR/Cas9 groups. Relative percentage of wound healed was calculated for the groups. Data represent mean (*n* = 4) ± SD. * = significantly different from NTC, *p* < 0.05. (D) % Cell migration by Boyden chamber technique. Liver cancer cells (Hep3B and HepG2) expressing either non‐targeting control (NTC) or PNO1 CRISPR/Cas9 were seeded. After 48 h of seeding, cell migration assays were performed as described in Section 2. Data represent mean (*n* = 4) ± SD. * = significantly different from control, and each other, *p* < 0.05. (E) % Cell invasion by Boyden chamber technique. Liver cancer cells (Hep3B and HepG2) expressing either non‐targeting control (NTC) or PNO1 CRISPR/Cas9 were seeded. After 72 h of seeding, cell invasion assays were performed as described in Section 2. Data represent mean (*n* = 4) ± SD. * = significantly different from control, and each other, *p* < 0.05.

Epithelial‐mesenchymal transition (EMT) is a process by which epithelial cells lose their polarity and adhesiveness, and gain migratory and invasive properties to become mesenchymal stem cells.^35^, ^36^ The EMT is associated with genetic changes that allow cells to leave the primary site and migrate to a secondary site to reestablish, differentiate, proliferate and survive.^37^ We next measured the influence of PNO1 knockout on cell motility. PNO1 knockout inhibited cell motility of both Hep3B and HepG2 cells (Figure 3A,B). PNO1 knockout significantly inhibited more than 55% of wound healed in both Hep3B and HepG2 groups (Figure 3C). The migration and invasion contribute to cancer progression and recurrence. Since knockout of PNO1 suppressed cell motility, we next measured the effects of PNO1 knockout on cell migration and invasion using Boyden chamber technique. PNO1 knockout inhibited cell migration and invasion of both Hep3B and HepG2 cells (Figure 3D,E). These data suggest that inhibition of PNO1 can be sufficient to suppress EMT and possibly dissemination of HCC cells.

### 3.4 PNO1 knockout modulates the expression of markers of epithelial‐mesenchymal transition in HCC

**FIGURE 4:**
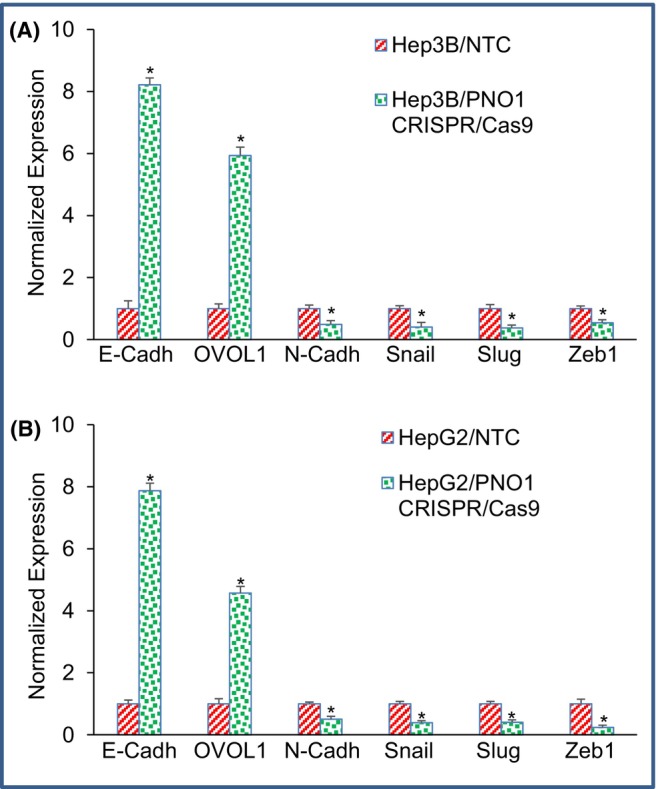
Effects of PNO1 knockout on the expression of EMT‐related genes in liver cancer cells. (A) Expression of EMT‐related genes in Hep3B cells. Liver cancer Hep3B cells expressing either non‐targeting control (NTC) or PNO1 CRISPR/Cas9 were seeded. After 24 h, cells were harvested, and RNA was extracted to measure the expression of E‐cadherin, OVOL1, N‐Cadherin, Snail, Slug and Zeb1 by qRT‐PCR. Data represent mean ± SD. * = significantly different from control, *p* < 0.05. (B) Expression of EMT‐related genes in HepG2 cells. Liver cancer HepG2 cells expressing either non‐targeting control (NTC) or PNO1 CRISPR/Cas9 were seeded. After 24 h, cells were harvested, and RNA was extracted to measure the expression of E‐cadherin, OVOL1, N‐Cadherin, Snail, Slug and Zeb1 by qRT‐PCR. Data represent mean ± SD. * = significantly different from control, *p* < 0.05.

Recurrence of cancer and metastasis are closely related to EMT.^38^, ^39^ EMT is characterized by the loss of epithelial markers, such as E‐cadherin, followed by upregulation of mesenchymal markers, such as N‐cadherin; resulting in promotion of cell migration and invasion of cancer cells as the initial step of metastasis.^37^ Transcription factors such as Snail, Slug and ZEB1 modulate the expression of cadherins and thus regulate EMT.^37^, ^40^ Since PNO1 knockout inhibited cell motility, migration and invasion, we next examined the molecular mechanisms of PNO1‐induced EMT regulation in HCC. As shown in Figure 4A,B, PNO1 knockout induced the expression of E‐cadherin and OVOL1 and inhibited the expression of N‐cadherin. Expression of epithelial and mesenchymal markers is tightly regulated by EMT transcription factors. Since PNO1 knockout modulated the expression of E‐cadherin, OVOL1 and N‐cadherin in Hep3B and HepG2 cells, we next sought to measure the expression of Snail, Slug and Zab1. PNO1 knockdown inhibited the expression of Snail, Slug and Zeb1 in Hep3B and HepG2 cells. Our findings suggest that PNO1 knockout can inhibit EMT by regulating the expression of cadherins, OVOL1, Snail, Slug and Zeb1 in HCC.

### 3.5 PNO1 knockout inhibits Notch signalling pathway in HCC

**FIGURE 5:**
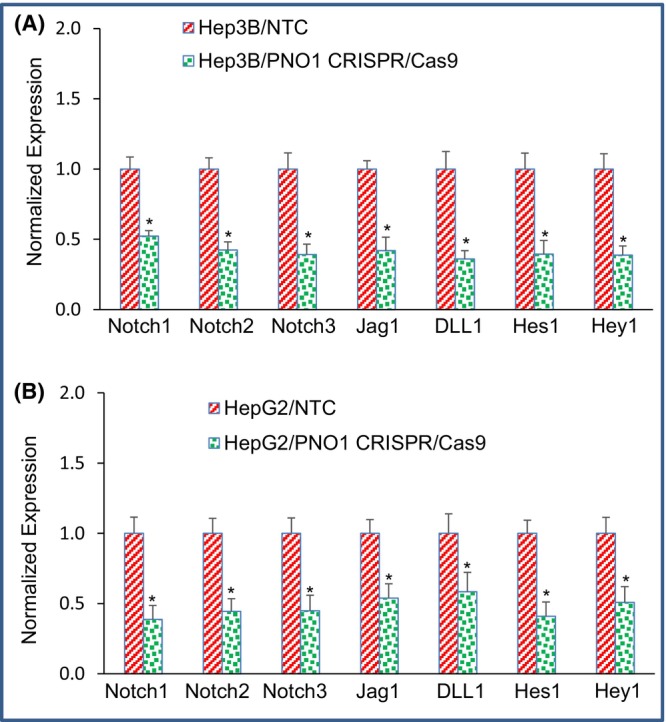
Effects of PNO1 knockout on Notch signalling pathway in liver cancer cells. (A) Expression of Notch pathway genes in Hep3B cells. Liver cancer Hep3B cells expressing either non‐targeting control (NTC) or PNO1 CRISPR/Cas9 were seeded. After 24 h, cells were harvested, and RNA was extracted to measure the expression of Notch1, Notch2, Notch3, Jagged1, DLL1, Hes1 and Hey1 by qRT‐PCR. Data represent mean ± SD. * = significantly different from control, *p* < 0.05. (B) Expression of Notch pathway genes in HepG2 cells. Liver cancer HepG2 cells expressing either non‐targeting control (NTC) or PNO1 CRISPR/Cas9 were seeded. After 24 h, cells were harvested, and RNA was extracted to measure the expression of Notch1, Notch2, Notch3, Jagged1, DLL1, Hes1 and Hey1 by qRT‐PCR. Data represent mean ± SD. * = significantly different from control, *p* < 0.05.

The pathological role of Notch signalling pathway in HCC is well established.^20^, ^41^ We sought to measure the effects of PNO1 knockdown on the components of Notch pathway and its target genes in Hep3B and HepG2 cells. PNO1 knockout inhibited the expression of Notch1, Notch2, Notch3 Jagged1 and DLL1 in HCC Hep3B and HepG2 cells (Figure 5A,B). Since inhibition of PNO1 regulated Notch pathway, we next sought to examine the expression of Notch‐target genes. PNO1 knockout inhibited the expression of Notch‐target genes Hes1 and Hey1 in both Hep3B and HepG2 cells. These findings suggest that PNO1 knockout can have a significant effect in inhibiting HCC growth by suppressing Notch pathway.

### 3.6 PNO1 knockout regulates genes involved in stemness, cell cycle and inflammation of HCC

**FIGURE 6:**
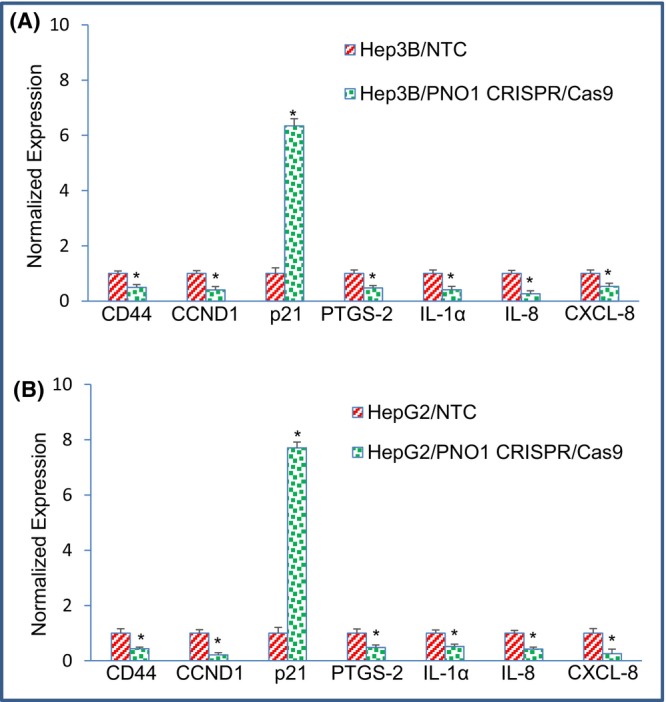
Effects of PNO1 knockout on the expression of genes involved in stemness, cell cycle, and inflammation in liver cancer cells. (A) Gene expression in Hep3B cells. Liver cancer Hep3B cells expressing either non‐targeting control (NTC) or PNO1 CRISPR/Cas9 were seeded. After 24 h, cells were harvested, and RNA was extracted to measure the expression of CD44, CCND1, p21, PTGS‐2, IL‐1α, IL‐8 and CXC‐8 by qRT‐PCR. Data represent mean ± SD. * = significantly different from control, *p* < 0.05. (B) Gene expression in HepG2 cells. Liver cancer HepG2 cells expressing either non‐targeting control (NTC) or PNO1 CRISPR/Cas9 were seeded. After 24 h, cells were harvested, and RNA was extracted to measure the expression of CD44, CCND1, p21, PTGS‐2, IL‐1α, IL‐8 and CXC‐8 by qRT‐PCR. Data represent mean ± SD. * = significantly different from control, *p* < 0.05.

Mounting evidence suggests that CD44 is a CSC marker and critical regulator of cancer stemness, including self‐renewal, tumour initiation and metastasis.^42^, ^43^ We sought to examine the effect of PNO1 knockout on the expression of CD44 (Figure 6A,B). PNO1 knockdown inhibited the expression of CD44 in both Hep3B and HepG2 cells. These data suggest that PNO1 knockout can inhibit HCC growth by targeting CSCs.

Activation or overexpression of oncogenes enhances cell division because most cell cycle regulator proteins are the products of oncogenes.^44^, ^45^ CCND1 gene acts at the G1/S stage of the cell cycle and is frequently overexpressed in cancer.^46^, ^47^, ^48^ P21 inhibits cell cycle progressions at G1/S and G2/M transition.^46^, ^47^, ^48^ We examined the effects of PNO1 inhibition on the expression of CCND1 and p21. PNO1 knockout inhibited the expression of CCND1 and upregulated the expression of cell cycle inhibitor p21 in Hep3B and HepG2 cells (Figure 6A,B). These data suggest that PNO1 can regulate cell cycle in HCC.

The expression of prostaglandin‐endoperoxide synthase (PTGS2, also known as cyclooxygenase 2) changes during inflammation and cancer initiation.^49^, ^50^ It plays a role in the prostanoid synthesis involved in apoptosis, cell differentiation and oncogenesis. We next examined the effects of PNO1 knockdown on the expression PTGS2 in HCC. PNO1 knockout inhibited the expression of PTGS2 in Hep3B and HepG2 cells (Figure 6A,B). These data suggest that inhibition of PNO1 can suppress inflammation in HCC.

Since PNO1 knockout inhibited the expression of PTGS2, a gene involved in inflammation, we, therefore, sought to examine the effects of inhibiting PNO1 on the expression of inflammatory cytokines (IL‐1α and IL‐8) and chemokine (CXCL‐8) in HCC cells. PNO1 CRISPR/Cas9 inhibited the expression of IL‐1α, IL‐8 and CXCL‐8 in Hep3B and HepG2 cells (Figure 6A,B). These data suggest that PNO1 knockout may inhibit inflammation by modulating the expression of inflammatory cytokines and chemokine in HCC.

### 3.7 PNO1 knockout inhibits colony formation, cell viability in spheroids and markers of stemness, pluripotency and EMT in CSCs

**FIGURE 7:**
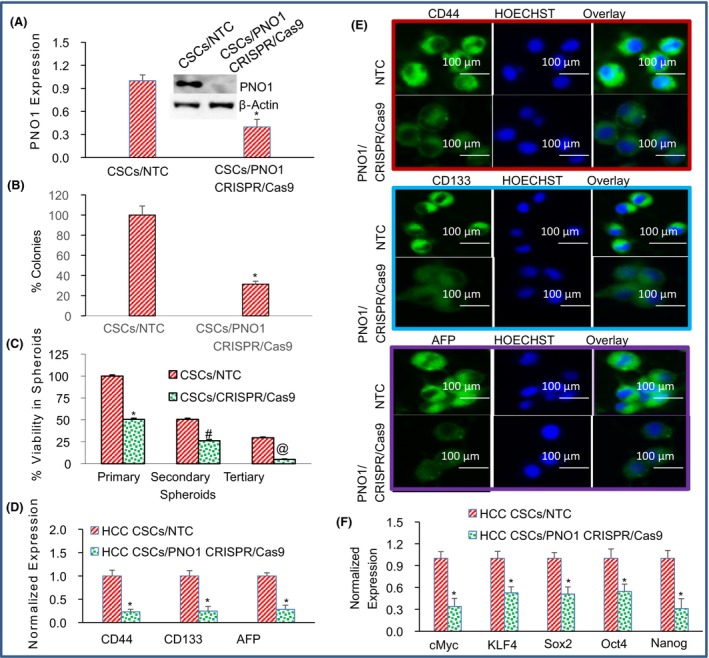
Effects of PNO1 knockout on cancer stem cell (CSC) characteristics. (A) PNO1 expression. CSCs were transduced with lentiviral particles expressing either non‐targeting control (NTC) or PNO1 CRISPR/Cas9, and the expression of PNO1 gene and protein was measured by qRT‐PCR and western blot analysis, respectively. Data represent mean ± SD. * = significantly different from control, *p* < 0.05. (B) % Colony formation. CSCs/NTC and CSCs/PNO1 CRISPR/Cas9 were seeded on dishes containing Matrigel. Number of colonies formed at 21 days were counted. Data represent mean (*n* = 4) ± SD. * = significantly different from NTC, *p* < 0.05. (C) % Cell viability in spheroids. CSCs/NTC and CSCs/PNO1 CRISPR/Cas9 were seeded on ultralow attachment plate in suspension for 7 days to obtain primary spheroids. At the end of incubation period, spheroids were collected and reseeded for another week to obtain secondary spheroids. At the end of second incubation period (2 weeks), spheroids were collected and reseeded for another week to obtain tertiary spheroids. Cell viability in spheroids was measured by trypan blue assay at the end of 7, and 14, 21 days. Data represent mean ± SD. *, #, and @ = significantly different from control, *p* < 0.05. (D) Expression of CD44, CD133 and AFP by qRT‐PCR. CSCs expressing either NTC or PNO1 CRISPR/Cas9 were seeded. After 24 h, cells were harvested, and RNA was extracted to measure the expression of CD44, CD133 and AFP by qRT‐PCR. Data represent mean ± SD. * = significantly different from control, *p* < 0.05. (E) Expression of CD44, CD133 and AFP by immunohistochemistry. CSCs expressing either NTC or PNO1 CRISPR/Cas9 were seeded. After 24 hrs, CSCs were fixed and the expression of CD44, CD133 and AFP was measured by immunohistochemistry. Green colour = CD44, CD133 or AFP. Blue colour = nuclei. (F) Expression of pluripotency‐maintaining factors (cMyc, KLF4, Sox2, Oct4 and Nanog). CSCs expressing either NTC or PNO1 CRISPR/Cas9 were seeded. After 24 h, CSCs were harvested, and RNA was extracted to measure the expression of cMyc, KLF4, Sox2, Oct4 and Nanog. Data represent mean ± SD. * = significantly different from control, *p* < 0.05.

Cancer stem cells have been shown to regulate cancer initiation, progression and metastasis in HCC.^42^, ^43^ We sought to assess the influence of PNO1 knockout on CSC's growth, spheroid formation and markers of stem cells, pluripotency and EMT. We inhibited the expression of PNO1 in HCC CSCs by CRISPR/Cas9 technology. CSCs were transduced with lentiviral particles expressing either non‐targeting (NTC) control or PNO1 CRISPR/Cas9, and the expressions of PNO1 gene and protein were measured by qRT‐PCR and western blot analysis, respectively. PNO1 knockdown inhibited the expressions of both PNO1 gene and protein in CSCs compared to NTC (Figure 7A).

Since PNO1 knockout inhibited PNO1 expression, we sought to examine the effects of PNO1 inhibition on colony formation in soft agar by CSCs. PNO1 knockout inhibited the number of colonies formed by CSCs compared to NTC (Figure 7B). The in vitro 3‐D culture system was developed to recapitulate the in vivo growth conditions of CSCs. The cancer 3‐D culture conditions preserve the biological characteristics of original tumours better than conventional 2‐D monolayer cultures. We therefore examined the effects of PNO1 inhibition on cell viability in spheroids (grown in suspension) formed by CSCs. PNO1 knockout inhibited CSC's viability in spheroids compared to NTC (Figure 7C). These data suggest that PNO1 knockout can inhibit CSC growth in HCC.

Cancer stem cells (CSCs) isolated from HCC are characterized by the expression of stem cell markers CD44, CD133 and alpha‐fetoprotein (AFP), and inhibition of these markers has been regarded as a reliable indicator for response to chemotherapy.^51^, ^52^, ^53^, ^54^ We therefore measured the effects of PNO1 inhibition on the expression of CD44, CD133 and AFP by qRT‐PCR (Figure 7D). PNO1 knockout inhibited the gene expression of CD44, CD133 and AFP in CSCs compared to NTC. We next measured the protein expression of CD44, CD133 and AFP by immunohistochemistry (Figure 7E). PNO1 knockdown inhibited the protein expression of CD44, CD133 and AFP in CSCs compared to NTC. These data suggest that PNO1 knockout can be effective in inhibiting CSC population in HCC.

CSCs exhibit the properties of pluripotency and self‐renewal which are maintained by cMyc, KLF4, Sox2, Oct4 and Nanog genes.^55^, ^56^ We measured the effects of PNO1 inhibition on the expression of cMyc, KLF4, Sox2, Oct4 and Nanog (Figure 7F). PNO1 knockout inhibited the expression of cMyc, KLF4, Sox2, Oct4 and Nanog compared to NTC. These data suggest that PNO1 knockout can be effective in the treatment of HCC by targeting CSCs.

**FIGURE 8:**
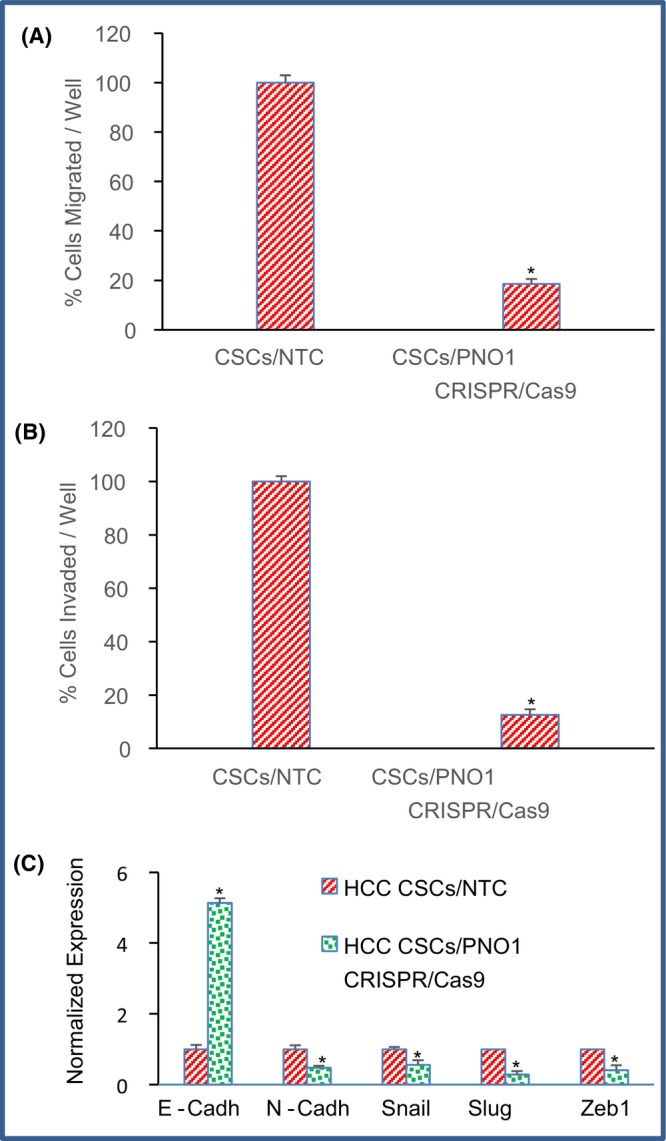
Effects of PNO1 knockout on migration, invasion and the expression of E‐Cadherin, N‐Cadherin, Snail, Slug and Zeb1 in CSCs. (A) % Cell migration. CSCs expressing either NTC or PNO1 CRISPR/Cas9 were seeded. After 48 h of seeding, cell migration assays were performed as described in Section 2. (B) % Cell invasion. CSCs expressing either NTC or PNO1 CRISPR/Cas9 were seeded. After 72 h of seeding, cell invasion assays were performed as described in Section 2. Data represent mean (*n* = 4) ± SD. * = significantly different from control, and each other, *p* < 0.05. (C) Expression of EMT‐related genes. CSCs expressing either NTC or PNO1 CRISPR/Cas9 were seeded. After 24 h, CSCs were harvested, and RNA was extracted to measure the expression of E‐cadherin, N‐Cadherin, Snail, Slug and Zeb1. Data represent mean ± SD. * = significantly different from control, *p* < 0.05.

We next measured the effects of PNO1 knockdown on cell migration and invasion because CSCs play a significant role in metastasis.^56^, ^57^ PNO1 knockdown inhibited cell migration and invasion of CSCs (Figure 8A,B). These data suggest that knockout of PNO1 by CRISPR/Cas9 can be sufficient to suppress EMT characteristics of CSCs. Since PNO1 knockout inhibited cell migration and invasion, we next examined the molecular mechanisms by which PNO1 knockout inhibited cell migration and invasion. PNO1 knockout induced the expression of E‐cadherin and inhibited the expression of N‐cadherin (Figure 8C). Expression of epithelial and mesenchymal markers is tightly regulated by EMT transcription factors. Since PNO1 knockout modulated the expression of cadherins in CSCs, we next sought to measure the expression of EMT‐related transcription factors (Figure 8C). PNO1 knockout inhibited the expression of Snail, Slug and Zeb1 in CSCs. These data suggest that PNO1 knockout can inhibit EMT characteristics by modulating the expression of cadherins, Snail, Slug and Zeb1.

## 4 DISCUSSION

In the present study, we found that PNO1 expression was upregulated in HCC samples and was associated with advanced TNM stages and lymph node metastasis. Knockout of PNO1 expression by the CRISPR/Cas9 technique suppressed HCC growth. PNO1 knockout inhibited liver cancer cell viability, colony formation and EMT and induced apoptosis. In addition to inhibiting the growth of liver cancer cells, PNO1 knockout also inhibited Notch signalling pathway, which has been shown to regulate cancer initiation, progression and metastasis. PNO1 knockout inhibited EMT by inducing a cadherin switch, up‐regulating OVO1 and suppressing the expression of EMT transcription factors (Snail. Slug and Zeb1). In addition to inhibiting cancer cells, PNO1 knockout inhibited CSC growth, migration and invasion, and also markers of stem cells, pluripotency and self‐renewal and EMT. Our findings suggest that PNO1 expression can be used as diagnostic/prognostic biomarker for HCC and inhibition of PNO1 expression by CRISPR/Cas9 technology can be a beneficial therapeutic strategy.

Ribosome biogenesis increases during malignant transformation and cancer progression and is positively correlated with protein synthesis.^58^, ^59^ We and others have found the oncogenic role of PNO1 in cancer.^7^, ^8^, ^9^, ^34^, ^49^, ^60^ Celecoxib, a nonsteroidal anti‐inflammatory drug, inhibited PNO1 expression and HCC tumour growth by regulating AKT/mTOR pathway.^60^ EBF1 overexpression down‐regulated PNO1 expression and transcription and upregulated the expression of p53 and p21 proteins in colorectal cancer.^61^ In another study, the downregulation of PNO1 expression inhibited the amounts of 18S rRNA, 40S subunits, 60S subunits and the 80S ribosome.^62^ Negatively regulated PNO1 expression by miR‐340‐5p promotes lung adenocarcinoma progression through Notch signalling pathway.^34^ Here, we showed that PNO1 expression in HCC increased with stage of development and nodal metastasis. Furthermore, HCC tissues with mutant Tp53 expressed higher PNO1 than those with wild‐type Tp53, suggesting the ability of transcription factor Tp53 to positively regulate PNO1 expression. We also demonstrated the overexpression of PNO1 in HCC and its knockdown by CRISPR/Cas9 technology inhibited cell proliferation, motility, migration and invasion. Overall, these data suggest that PNO1 acts as an oncogene and can also serve as a diagnostic and prognostic biomarker for HCC.

CSCs possess high capacity for self‐renewal, differentiation and tumorigenesis, and therefore are considered as attractive target for eradicating HCC. CSCs are generated under inflammatory conditions through a process of malignant transformation.^63^ Liver CSCs can be generated by di‐differentiation of hepatocytes. During this process, transformed cells gain the phenotype of stem cells by expressing stem cell markers and pluripotency‐maintaining factors.^63^, ^64^, ^65^, ^66^, ^67^ CSCs are generally more tumorigenic than other non‐stem cancer cells and resistant to multiple anticancer therapies, including chemotherapy and radiotherapy.^68^ HCC with stemness‐related marker expression has been associated with increased serum AFP levels and a poor prognosis.^69^ In the present study, PNO1 knockdown inhibited stem cell markers (CD44, CD133 and AFP) and pluripotency‐maintaining factors (cMyc, KLF4, Oct4 and Sox2 and Nanog). These data suggest that PNO1 inhibition can be beneficial for the treatment of HCC by targeting CSCs.

The Notch signalling pathway regulates liver development, differentiation and regeneration post‐injury.^20^, ^41^ Aberrant activation of the Notch pathway has been associated with HCC development.^20^, ^41^ We showed that PNO1 knockout inhibited Notch signalling by reducing the expression of Notch receptors, their ligands and also downstream targets. Our study is in an agreement with other where PNO1 enhanced HCC progression by modulating Notch signalling pathway.^34^ Overall, these data suggest that PNO1 expression by CRISPR/Cas9 technique can exploited to regulate HCC development.

The EMT is a highly dynamic process in carcinogenesis, involving the disruption of cell–cell adhesion and cellular polarity, remodelling of the cytoskeleton and changes in cell–matrix adhesion.^36^, ^70^ Mesenchymal‐epithelial transition (MET) is a reversal of EMT, where circulating cancer cells, after reaching a desirable metastatic niche, repolarize to colonize and develop secondary tumours.^36^, ^70^ Switch in gene expression from epithelial to mesenchymal phenotype is triggered by complex regulatory networks involving transcriptional control with Snail, Slug and Zeb1.^71^ In the present study, PNO1 knockout inhibited EMT by inducing a cadherin switch, up‐regulating OVOL1, and inhibiting Snail, Slug and Zeb1 in HCC. Therefore, EMT modulation by PNO1 knockout will inhibit metastasis in addition to tumour growth. However, more knowledge about the role of PNO1 on EMT and metastasis is necessary.

Ribosome biogenesis modulates the expression of genes which directly regulate cell cycle. Impaired ribosome biogenesis has been shown to inhibit cell cycle progression.^72^ The p53 signalling pathway has been shown to link ribosome biogenesis and the cell cycle.^73^ Overexpression Myc in transgenic mice model showed the significance of wild‐type p53 in suppressing cell proliferation and tumour growth after obstructed ribosome biogenesis.^73^, ^74^ In other studies, the inhibition of ribosome biogenesis caused cell cycle arrest in a p53‐independent manner.^75^, ^76^ Here, we showed that PNO1 knockout inhibited p21 and suppressed cyclin D1 in HCCs. Our data and others have clearly demonstrated a link between ribosome biogenesis and cell cycle progression in both p53‐dependent and independent manners.

Chronic inflammation is a risk factor for the onset of cancer and is associated with an enhanced ribosome biogenesis.^77^, ^78^ Elevated levels of prostaglandins, inflammatory cytokines and chemokines have been linked with inflammation.^79^, ^80^, ^81^, ^82^ The induction of inflammatory signals causes cancer initiation in more than 90% of HCC cases. Here, we showed that PNO1 knockout inhibited genes involved in surface adhesion (CD44), cell cycle regulation (CCND1 and p21), inflammation (PTGS‐2, IL1a and IL‐8) and chemotaxis (CXCL‐8) of HCC. Overall, these data suggest that the involvement of inflammatory cytokines and chemokines during PNO1 regulated HCC initiation and progression.

Collectively, the preclinical data reported herein provide a promising strategy for treating HCC patients whose tumours harbour higher PNO1 expression and constitutively active Notch signalling pathway. Although the direct mechanistic link between PNO1 inhibition and gene/pathway regulation could not establish in our studies, knockout of PNO1 expression by CRISPR/Cas9 technique might prove beneficial for treating HCC patients. Furthermore, the strategy will also be useful in those HCC patients whose cancer relapse because inhibition of PNO1 expression suppresses CSC characteristic.

## AUTHOR CONTRIBUTIONS

**Sanjit K. Roy:** Conceptualization (equal); data curation (equal); formal analysis (equal); methodology (equal); validation (equal); visualization (equal); writing – original draft (equal); writing – review and editing (equal). **Shivam Srivastava:** Conceptualization (equal); data curation (equal); formal analysis (equal); methodology (equal); visualization (equal). **Caroline McCance:** Visualization (equal); writing – review and editing (equal). **Anju Shrivastava:** Formal analysis (equal); methodology (equal); writing – review and editing (equal). **Jason Morvant:** Conceptualization (equal); methodology (equal); writing – review and editing (equal). **Sharmila Shankar:** Formal analysis (equal); writing – review and editing (equal). **Rakesh K. Srivastava:** Methodology (equal); resources (equal); supervision (equal); writing – review and editing (equal).

## CONFLICT OF INTEREST STATEMENT

All the authors have declared that no competing interests exist.

## Data Availability

The data that support the findings of this study are available from the corresponding author upon reasonable request.
